# Barriers and facilitators to HIV viral load suppression among adolescents living with HIV in Lubumbashi, Democratic Republic of the Congo: A qualitative study

**DOI:** 10.1371/journal.pone.0320417

**Published:** 2025-03-25

**Authors:** Olivier Mukuku, Kaymarlin Govender, Stanislas Okitotsho Wembonyama

**Affiliations:** 1 Department of Maternal and Child Health, Institut Supérieur des Techniques Médicales de Lubumbashi, Lubumbashi, Democratic Republic of the Congo,; 2 Health Economics and HIV and AIDS Research Division, University of KwaZulu-Natal, Durban, KwaZulu-Natal, South Africa,; 3 Department of Pediatrics, Faculty of Medicine, University of Lubumbashi, Lubumbashi, Democratic Republic of the Congo; Institute of Tropical Medicine / University of Antwerp, BELGIUM

## Abstract

**Background:**

Antiretroviral therapy (ART) has been pivotal in improving the lives of adolescents living with HIV (ALHIV) globally. However, achieving and maintaining viral load suppression (VLS) among ALHIV remains a significant challenge, with difficulties reported in various settings, including places like Lubumbashi, in the Democratic Republic of the Congo. Despite the availability of ART, several barriers to optimal ART adherence and achieving VLS persist among this population. This qualitative study aimed to explore the factors influencing ART adherence and achieving VLS among ALHIV in Lubumbashi.

**Methods:**

In-depth interviews were conducted with 39 ALHIV (22 female, 17 male) receiving ART and 14 caregivers (9 female, 5 male) participating in their treatment. ALHIV were purposively selected based on criteria including being HIV-positive, on ART, informed of their HIV status, and aged 13-19 years. Caregivers were purposively sampled based on their involvement in the treatment and care of the ALHIV. Focus group discussions were held with 16 healthcare workers (HCWs) (10 female, 6 male), including doctors, nurses, and pharmacists, who had at least one year of experience caring for ALHIV in the selected clinics. The sessions were audio-recorded, transcribed, and analyzed using a thematic approach to identify recurring themes and patterns. Data analysis was guided by the socioecological model, examining factors at the individual, interpersonal, health service-related, and community levels. NVivo 14 software was used for data management and analysis.

**Results:**

The key barriers to achieving VLS identified included economic challenges, stigma and discrimination, forgetfulness, and a lack of family support. Factors such as strong social support, regular counseling, use of reminder tools, and positive HCW-patient relationships were found to facilitate ART adherence and achieving VLS. The participants emphasized the importance of addressing psychosocial challenges alongside medical treatment to improve outcomes for ALHIV.

**Conclusions:**

Improving VLS among ALHIV in Lubumbashi requires a multifaceted approach that includes strengthening family and community support systems, simplifying ART regimens, and enhancing HCW capacity to offer comprehensive care. Policy interventions and collaborative efforts across sectors are essential to overcoming the barriers to ART adherence and achieving sustained VLS in this vulnerable population.

## 1. Introduction

The global health community has relentlessly pursued progress in the ongoing efforts to address human immunodeficiency virus (HIV), with the advent and optimization of antiretroviral therapy (ART) testifying to this commitment. Significant progress has been made in the global response to HIV/AIDS, mainly owing to the widespread implementation of ART [1]. The main objective of ART is to achieve viral load suppression (VLS) to undetectable levels, a step that not only improves the health and longevity of people living with HIV (PLHIV) but also eliminates the risk of viral transmission to others, as demonstrated by the Partner Study [2]. By 2025, to meet the UNAIDS 95-95-95 goal of halting the HIV transmission, it is essential to ensure that 95% of PLHIV know their serostatus, 95% of those diagnosed with HIV receive continuous ART, and 95% of those on ART achieve VLS [3]. Current data indicate that a large majority of PLHIV achieve VLS within six months of starting ART, provided that they adhere strictly to their ART regimen [4,5]. In cases where VLS is not achieved within the first six months of ART, seven out of ten PLHIV are able to achieve VLS later on with enhanced support for ART adherence [6]. The World Health Organization (WHO) recommends in-depth counseling on ART adherence for individuals with an unsuppressed viral load (VL) [7]. ART has transformed HIV from a fatal diagnosis into a manageable chronic disease, yet achieving VLS remains a challenge, particularly for adolescents living with HIV (ALHIV). Failure to meet the UNAIDS 95-95-95 targets for children and adolescents in sub-Saharan Africa (SSA), home to 90% of children living with HIV, risks sustaining new infections and HIV-related mortality [8]. This challenge is compounded by the unique developmental issues adolescents face, which may exacerbate the difficulties in managing HIV [9]. Adolescence, a time of psychological and emotional development, is a period when individuals navigate identity formation and complex social dynamics. ALHIV face distinct barriers to ART adherence, often resulting in virological failure and poorer treatment outcomes compared to adults [10,11]. These barriers are particularly pronounced in low- and middle-income countries, where limited healthcare infrastructure, economic constraints, and lack of psychosocial support complicate treatment adherence. Studies such as Audi et al. [12] have shown the positive impact of peer support groups on ART adherence by enhancing knowledge and confidence. However, barriers remain, including the burden of pills, fear of stigma, and lack of resources [13,14]. Researches by Gordon et al. [1] and Izudi et al. [15] have emphasized that achieving VLS requires addressing not just medical but also social and personal factors, including undisclosed HIV status, broken families, stigma, and noncompliance with ART guidelines.

Data on VLS among ALHIV are scarce in the Democratic Republic of the Congo (DRC). Moreover, studies in SSA have shown varying rates of VLS among ALHIV across countries, ranging from 21.15% in Cameroon (Douala) [16] to 32.55% in Kenya [17], 35.05% in Zimbabwe [18], 38.76% in Malawi [19], and 54.08% in Cameroon (Yaoundé) [20]. These challenges are not unique to the DRC but are pervasive in many countries and regions, including urban areas like Lubumbashi, where ALHIV face unique obstacles in accessing comprehensive care and support services due to limited health facility access, stigma, and discrimination [21]. Despite efforts to expand ART coverage, achieving VLS in ALHIV remains a significant challenge in the country. This study aims to investigate the barriers and facilitators of ART adherence and VLS achievement among ALHIV aged 13-19 years in Lubumbashi, in the DRC. By identifying the specific contextual factors influencing ART adherence and VLS achievement, this study seeks to inform the development of targeted interventions to improve HIV care and support services for adolescents in the region.

## 2. Materials and methods

### 2.1. Study design and setting

This qualitative study was conducted at five HIV care clinics in Lubumbashi, in the DRC, from June 10 to July 31, 2024. Lubumbashi, the second-largest city in the DRC, is located in the southeastern part of the country. The city has an estimated population of over 2,933,962 in 2024, covering an area of 747 km² and resulting in a population density of approximately 3,927 inhabitants per km² [22]. Healthcare facilities were selected based on their capacity to provide HIV care specifically for adolescents living with HIV, with no consideration given to the number of PLHIV, facility type (public or private), or ownership, as all selected facilities receive similar support through the Haut-Katanga provincial health authority, the U.S President’s Emergency Plan for AIDS Relief (PEPFAR) funding, and coordination by the DRC National HIV/AIDS Programme.

### 2.2. Study population

The study triangulated data from different sources, including ALHIV (aged 13–19 years) and their caregivers through in-depth interviews (IDIs), and healthcare workers (HCWs) through focus group discussions (FGDs). A purposive sampling method was used to select participants based on specific inclusion criteria. Initially, ALHIV were identified from the clinical records and registers at the HIV care clinics in Lubumbashi, with permission obtained from the relevant ethical committees and health facilities to access and review these records. This process allowed us to ensure that only those who met the inclusion criteria were selected.

To be eligible for inclusion in the study, adolescents had to meet the following criteria: (1) being HIV-positive, (2) currently receiving ART for a minimum of 6 months, (3) residing in Lubumbashi city, (4) aware of his/her HIV status, and (5) having undergone VL testing, with classification as either virally suppressed (VL <  1,000 copies/mL) or unsuppressed (VL ≥  1,000 copies/mL) in line with the DRC National HIV/AIDS Programme and WHO guidelines. Additionally, participants needed to provide informed consent or assent to join in the study. Adolescents not on ART or who had not initiated treatment yet, as well as those on ART for less than six months, were excluded from the study.

We systematically selected participants who met these criteria and ensured that we also included caregivers of the ALHIV. The goal was to achieve a balanced and comprehensive understanding of the barriers and facilitators to ART adherence and to achieve VLS among this group. Given that younger adolescents rely on their parents or guardians for ART adherence and treatment decisions, we also purposively sampled adult parents or guardians who agreed to participate and provided written informed consent. Parents or guardians of ALHIV who were severely ill and in need of immediate care at the time of the study were excluded.

Additionally, healthcare workers, including doctors, nurses, and pharmacists involved in the care of ALHIV in the health facilities selected for the study were purposively selected to participate in FGDs. These HCWs were chosen to participate in FGDs because of their expertise in managing ALHIV via ART and their understanding of the challenges related to ART adherence and the specific care needs of this population. We included those with one or more years of experience in the care of ALHIV, excluding those who had been newly transferred to the study site for less than six months. A purposive sampling technique was used to select participants based on characteristics or traits that would allow for a detailed understanding of the subject. Selection decisions were based on research questions, theoretical perspectives, and evidence informing the study.

In our study, PLHIV were primarily cared for through the HIV care clinics in Lubumbashi, where they received ART and ongoing health support. PLHIV, including the ALHIV, were part of routine care provided by HCWs such as doctors, nurses, and pharmacists at these clinics. The care provided to PLHIV included regular ART follow-ups, VL testing, and psychosocial support to help manage both the physical and emotional aspects of living with HIV. HCWs were trained and experienced in managing the healthcare needs of ALHIV, with a particular focus on ART adherence and VLS. This healthcare support was part of the broader HIV care services facilitated through national and international programs such as PEPFAR. In this study, ALHIV and their caregivers, were involved through their participation in interviews. This provided insights into the specific challenges they faced in managing HIV, particularly regarding ART adherence, psychosocial barriers, and support systems. Their involvement in the study did not interfere with their ongoing care (without disrupting their regular care routines), and all interviews were conducted in a manner that respected their confidentiality and well-being.

### 2.3. Conceptualization and theoretical framework

Data from this study were interpreted through the analytical lens of the socioecological model which contends that an’individual’s behavior is shaped by factors at various levels, including individual (intrapersonal), relationship (interpersonal), health service-related, and community levels [23]. Intrapersonal factors included attributes like age, gender, education, behavior, and attitude. Interpersonal factors referred to influences from close circles including friends, peers, and families. Health service-related factors covered health system aspects such as access to health services, quality of health education, referrals, worker training, and staffing [23]. Community factors involved stigma, discrimination, community beliefs, and cultural norms related to HIV.

### 2.4. Data collection process

The IDIs and FGDs were conducted using interview guides customized for each participant group, featuring open-ended questions, and conducted in either French or Swahili based on the participants’ language preference. The interview guides used for both the IDIs and FGDs were designed based on the socioecological model, which served as the theoretical framework for our study. This model helped structure the questions to explore the various factors influencing ART adherence and VLS among ALHIV. The questions were developed to capture perspectives at multiple levels, including individual, interpersonal, health service-related, and community factors. Additionally, the guides incorporated insights from the literature on HIV care, ART adherence, and the psychosocial challenges faced by ALHIV. The sessions were recorded and subsequently translated into English for analysis.

Thirty-nine ALHIV who met the inclusion criteria were identified from the clinical records of HIV care clinics. Each ALHIV was interviewed individually. HCWs from selected HIV care clinics had previously reached out to the ALHIV and their caregivers to arrange appointments. Four FGDs were carried out with 16 HCWs, with each group consisting of a doctor, two nurses, and a pharmacist.

The interviews were conducted in private and quiet rooms within the clinics where the ALHIV received care, from Monday to Friday between 9:00 a.m. and 4:00 p.m., with an average duration of 25-30 minutes per interview. Throughout the data collection process, the principal investigator (O.M.) was assisted by two clinical psychologists, each with over seven years of experience in providing care for PLHIV. These psychologists were recruited and underwent training on various topics, including the study’s objectives, qualitative research techniques, participant recruitment, data management, research ethics, and quality assurance. Additionally, they received specialized training on conducting interviews with vulnerable populations, specifically ALHIV. The IDIs utilized a semi-structured guide with open-ended questions and suggested prompts that covered a range of topics such as HIV knowledge, treatment, factors influencing VLS and ART adherence, psychosocial support, personal perspectives on medication, and barriers and facilitators to achieving VLS and ART adherence for ALHIV.

### 2.5. Data processing and analysis

The audio recordings from the FGDs with HCWs and the IDIs with ALHIV and their caregivers were meticulously transcribed verbatim and then imported into NVivo 14 software for analysis. To obtain a comprehensive understanding of the data, two researchers (O.M. and S.O.W.) first examined the transcripts in their entirety, allowing them to develop preliminary coding frameworks. Separate coding frameworks were created for the interviews with ALHIV and caregivers, as well as for the FGDs with HCWs, considering the variations in the study populations and data collection techniques. The discussion topics were structured around the socioecological model, which posits that an individual’s behavior is shaped by factors operating at various levels, including individual, family/social/interpersonal, health service-related, and community levels.

Next, a thorough comparative content analysis was carried out, involving a re-examination of each transcript and the assignment of codes to corresponding thematic sections. While some codes were predefined, others surfaced as novel themes. The text was subsequently arranged according to these codes and consolidated into matrices to pinpoint prevalent concerns and variations in the narratives. Throughout the analysis process, discussions were held to tackle and reconcile any discrepancies in coding.

To ensure data saturation and capture emerging or divergent themes, the data were reviewed periodically throughout the study. After the initial interviews and FGDs, the data were revisited regularly to identify new themes as they emerged, allowing for an iterative approach to analysis.

Additionally, field notes were utilized throughout the data collection process. The principal investigator (O.M.) and the clinical psychologists involved in data collection meticulously documented field notes during the IDIs and FGDs. These notes encompassed contextual details, non-verbal cues, and reflections on the interview dynamics. The field notes were cross-referenced with the transcriptions to enrich the analysis and gain a more holistic insight into the outcomes.

The conceptualization and analysis of the data were further informed by the socioecological model, which aided in comprehending the dynamic interactions between ALHIV and their environment. Once the data were organized into matrices and consolidated into broad themes, those specifically related to ART adherence were aligned with an ecological systems model to better illustrate the findings.

### 2.6. Ethical statements

The study received approval from several key bodies: the Medical Ethics Committee of the University of Lubumbashi (N° UNILU/CEM/036/2023), the Humanities & Social Sciences Research Ethics Committee of the University of KwaZulu Natal (N° HSSREC/00006817/2024), the Haut-Katanga Provincial Ministry of Public Health (N° 10.8/001257/CAB/MIN.PROV/SANTE&C.O.NU/HKAT/2023 and N°10.8/002725/ CAB/MIN.PROV/SANTE&C.O.NU/HKAT/2024), and the managers of HIV care clinics. These entities thoroughly reviewed and approved the study protocol, research instruments, and procedural methods before fieldwork commenced. The study adhered to the ethical standards for research involving human subjects as defined by the DRC and the 1964 Declaration of Helsinki, including its subsequent amendments.

Written informed assent was obtained from ALHIV, while written informed consent was provided by their parents or biological family members. When the guardian was not a biological family member, consent was obtained from a legal guardian. During the in-depth interviews, consent was also obtained from the HCW and their parents or guardians. ALHIV and their caregivers received a snack and transportation stipends of $5 USD each. The transportation stipend was provided to cover travel expenses, while the snack was offered as a gesture of appreciation for their time and participation. Both the stipend and the snack were intended to support participants in their involvement in the study and were not intended to influence their decision to participate. HCWs were compensated for their time and contributions to the study.

## 3. Results

### 3.1. Characteristics of the study participants

Table 1 presents the characteristics of the ALHIV, their parents/guardians and the HCWs involved in the study. The majority of ALHIV were aged between 16 and 19 years (35/39) and the majority were female (22/39). The majority of ALHIV (33/39) had a suppressed HIV VL and were in secondary school (35/39). In terms of living arrangements, more than half of the adolescents lived with at least one biological parent (20/39), whereas 28.2% lived with non-parental family members. The caregivers were mainly aged between 40 and 59 years (6/14) and were mostly women (9/14). The HIV status of these caregivers varied: just over one-third were HIV-positive (5/14), whereas 42.9% (6/14) did not know their HIV status. Most of the HCW were women (10/16), and most were aged 40 years or over (9/16). The group comprised 4 doctors, 8 nurses, and 4 pharmacists.

**Table 1:** Characteristics of the study participants.

Adolescents	N = 39
Age	
13 - 15 years	4 (10.3%)
16 - 19 years	35 (89.7%)
Mean ± standard deviation	17.14 ± 1.06
Sex	
Female	22 (56.4%)
Male	17 (43.6%)
HIV viral load status	
Suppressed	33 (84.6%)
Non-suppressed	6 (15.4%)
Educational level	
Secondary	35 (89.7%)
Higher	4 (10.3%)
Occupation	
Student	34 (87.2%)
Self-employed	5 (12.8%)
Currently living with/in	
At least one biological parent	20 (51.3%)
A non-parent family member	11 (28.2%)
A non-family member	5 (12.8%)
Group housing	3 (7.7%)
Current ART regimen	
Dolutegravir based regimen	36 (92.3%)
Efavirenz based regimen	3 (7.7%)
**Caregivers**	**N = 14**
Age	
20-39 years	4 (28.6%)
40-59 years	6 (42.8%)
≥60 years	4 (28.6%)
Mean ± standard deviation	47.21 ± 12.26
Sex	
Female	9 (64.3%)
Male	5 (35.7%)
HIV status	
Positive	5 (35.7%)
Negative	3 (21.4%)
Unknown	6 (42.9%)
**Healthcare workers**	**N = 16**
Age	
20-39 years	7 (43.8%)
≥40 years	9 (56.2%)
Mean ± standard deviation	41.75 ± 13.17
Sex	
Female	10 (62.5%)
Male	6 (37.5%)
Medical title	
Doctor	4 (25.0%)
Nurse	8 (50.0%)
Pharmacist	4 (25.0%)

### 3.2. Barriers to and facilitators of viral load suppression in ALHIV according to the socioecological model

Table 2 below summarizes the barriers and facilitators to achieve VLS among ALHIV aged 13-19 years receiving ART in Lubumbashi, in the DRC, based on the domains of the socioecological model.

**Table 2:** Summary of barriers and facilitators to achieve viral load suppression in ALHIV according to the socioecological model.

Level	Barriers	Facilitators
**Individual**	• ART side effects • Forgetting to take medication • Lack of food • Being too busy • Feeling healthy and not perceiving the necessity of ART • Being away from home	• Confidence in the effectiveness of ART • Awareness of the importance of ART adherence • Having a structured routine • Use of reminder tools • Ability to manage side effects of ART
**Interpersonal**	• Undisclosed HIV-positive status	• Staying healthy for his/her loved ones • Peer support clubs
**Family**	• Broken families and loss of parents • Lack of assistance in taking ART • Stigma and discrimination	• Support from family and friends in initiating and adhering to ART
**Health service**	–	• Positive and open relations with nursing staff • Mental health support
**Community**	• Stigma and discrimination	–

### 3.3. Facilitators of viral load suppression

#### 3.3.1. Individual facilitators.

##### 3.3.1.1. Confidence in the effectiveness of ART

ALHIV and their caregivers expressed growing confidence in the effectiveness of ART, which fosters strict adherence to treatment. This belief in the advantages of ART boosts their commitment to consistent medication intake, supported by caregivers throughout the journey.

*“Since I have been taking my ART daily, my life has completely changed. My condition has stabilized, my VL is undetectable, and I can live a full life despite my HIV status.”* (Adolescent 32, female, 16 years)

*“My two children and I are HIV-positive. I always take my ART at the same time as my children to stay healthy and live a long life.”* (Caregiver 13, mother, HIV-positive)

##### 3.3.1.2. Awareness of the importance of ART adherence

Understanding the critical role of adherence to ART has empowered ALHIV and caregivers to prioritize treatment. This awareness often stems from personal experiences and psychological support, reinforcing the necessity of consistent adherence.

*“When my daughter learned her medication was for HIV, she stopped taking it. I sought advice from psychologists and constantly reminded her about the risks. Now, she takes it on her own and keeps her VL under control.”* (Caregiver 04, father, HIV-positive)

*“I thought I could manage without ART, but my health worsened drastically. From that moment, I vowed to follow my treatment rigorously.”* (Adolescent 34, female, 19 years)

##### 3.3.1.3. Importance of a structured routine

A structured daily routine emerged as a crucial factor for maintaining ART adherence and achieving VLS. Participants emphasized the role of discipline and consistency in promoting successful health management.

*“Since I was young, I’ve taken my medicine at 8:30 p.m. without reminders. Even when traveling, I never miss it.”* (Adolescent 05, male, 19 years)

*“My daughter and I are both HIV-positive. We remind each other to take our medication daily, even from a distance.”* (Caregiver 11, mother, HIV-positive)

##### 3.3.1.4. Use of reminder tools

Reminder tools such as alarms, diaries, and mobile phones have significantly reduced forgetfulness and enhanced adherence to ART. Family support also plays a vital role, particularly when an adult ensures medication intake.

*“I bought a small phone for a 16-year-old girl in my care. The alarm reminds her to take her medication, even during challenging times like funerals.”* (Caregiver 05, non-family member, HIV unknown status)

##### 3.3.1.5. Managing ART side effects

Building resilience to manage ART side effects is crucial for adherence and achieving VLS. Caregivers and HCWs offer counseling to empower ALHIV and their families to overcome challenges caused by nausea, fatigue, and other side effects.

*“Despite experiencing nausea and fatigue, I remind myself of the significance of maintaining my health. As advised by medical professionals, I ensure to have a meal before taking my medication.”* (Adolescent 24, female, 17 years)

*“Since transitioning to TLD [Tenofovir + Lamivudine + Dolutegravir], we’ve observed fewer ART side effects compared to previous regimens.”* (FGD 01, HCW 04, male, doctor)

#### 3.3.2. Interpersonal facilitators.

##### 3.3.2.1. Staying healthy for their loved ones

Emotional connections with loved ones serve as a powerful motivator for ART adherence and achieving VLS among ALHIV. Support from family and significant others fosters a sense of responsibility and encourages ALHIV to remain committed to their treatment, even during challenging times.

*“Since I started dating my girlfriend, my commitment to ART has grown. Even when feeling discouraged, her encouragement and my mother’s reminders help me stay determined.”* (Adolescent 19, male, 18 years)

These relationships are crucial, underscoring how family and close connections significantly influence treatment outcomes.

##### 3.3.2.2. Peer support clubs

Peer support clubs, especially those on platforms like WhatsApp, significantly improve ART adherence among ALHIV. These groups foster a sense of community and mutual accountability, inspiring members to adhere to their medication schedules and exchange coping strategies.

*“Someone in our JADO WhatsApp group asks if everyone has taken their medication. This reminder helps me stay on track.”* (Adolescent 34, female, 19 years)

Peer support is particularly transformative for younger adolescents struggling with their diagnosis.

*“My 12-year-old niece struggled with her diagnosis. After joining a support group, she became more consistent with her ART and successfully suppressed her VL.”* (Caregiver 10, maternal uncle, HIV unknown status)

HCWs highlight the importance of peer educators in facilitating these groups:

*“Peer educators assist ALHIV in engaging in support groups, where they share experiences and motivate each other to enhance ART adherence and achieve VLS. Some have established WhatsApp groups to enhance this support.”* (FGD 02, HCW 03, male, pharmacist)

These clubs foster resilience, normalize challenges, and create a positive environment that reinforces adherence.

#### 3.3.3. Family facilitators: Support from family and friends in initiating and adhering to ART.

Family and friend support is crucial for achieving VLS among ALHIV. This support provides emotional encouragement and practical assistance, including reminders to take medication and attend medical appointments. Caregivers play a significant role in assisting adolescents in navigating the emotional challenges of an HIV diagnosis. Often, ALHIV may experience denial or despair initially, which can hinder adherence. Therefore, ongoing support from caregivers is essential in fostering commitment to treatment.

*“I used to consider giving up, but my mother and aunt motivate me daily. Their support helps me stay disciplined with my medication, knowing I’m important to them and they prioritize my well-being.”* (Adolescent 28, female, 19 years)

Shared family routines can reinforce adherence.

*“My 18-year-old daughter and I are both HIV-positive. We take our medication together at 8 p.m. each night, ensuring one of us remembers if the other forgets.”* (Caregiver 09, mother, HIV-positive status)

A caregiver involvement in healthcare appointments also enhances adherence:

*“My 16-year-old daughter refused her medication and appointments. However, since I started accompanying her, she now consistently attends appointments, and we take our medication together.”* (Caregiver 12, mother, HIV-positive status)

#### 3.3.4. Health service facilitators.

##### 3.3.4.1. Positive and friendly relationships with HCWs

In the global response to HIV, constructive and supportive relationships with HCWs play a critical role in achieving VLS among ALHIV. These interactions help adolescents understand their condition and the importance of ART adherence, thereby reducing the risk of treatment interruption and unsuppressed VL. Positive relationships foster open communication, enabling ALHIV to seek guidance and address challenges effectively.

*“I have a very good relationship with my doctor; he advises and encourages me to take my medication... When I meet with him, he listens to my concerns, discusses various topics, and provides valuable feedback on my health. I truly feel cared for.”* (Adolescent 39, female, 17 years)

##### 3.3.4.2. Psychological support

ALHIV often face stigma and discrimination, which can lead to significant psychological distress, undermining ART adherence and VLS achievement. Health services stress the importance of psychological support from families, friends, and professionals to assist adolescents in coping with stigma-related stress and depression.

*“Most ALHIV struggle to accept their status after prolonged medication use without disclosure. When they learn their HIV-positive status, many experience despair and psychological issues, leading to missed medical appointments. To address this, we collaborate with clinical psychologists to provide HIV education and mental support. This helps them adhere to ART and regain emotional stability.”* (FGD 03, HCW 05, female, nurse)

### 3.4. Barriers to viral load suppression

#### 3.4.1. Individual-level barriers.

##### 3.4.1.1. ART side effects

ART side effects, such as nausea, headaches, and fatigue, are significant barriers to ART adherence and achieving VLS among ALHIV. These discomforts often deter consistent treatment, despite the efforts of caregivers to provide support.

*“Before starting treatment, I felt fine and didn’t even know I was HIV-positive. However, since I started treatment, I have been constantly suffering from nausea, headaches, and fatigue, which makes it difficult to follow the treatment rigorously and keep my VL under control. Sometimes these side effects discourage me and seem insurmountable.”* (Adolescent 35, male, 18 years)

##### 3.4.1.2. Forgetting to take medication

Forgetting to take medication is a common issue. Life occupations/distractions such as school, social activities, or work can interfere with adherence, impacting VLS.

*“Sometimes I’m in a hurry to get to mass as a singer in the choir, and since the church is quite far away, I go home exhausted and forget to take my medicine.”* (Adolescent 02, female, 16 years)

*“... on several occasions I’ve gone to parties and had beer. When I got home, I forgot to take my medication because I was so drunk.”* (Adolescent 35, male, 18 years)

##### 3.4.1.3. Lack of food

A lack of food is a significant barrier to ART adherence for some ALHIV, particularly those living in precarious situations, such as orphans with limited financial resources. This scarcity of food can make it challenging to adhere to medication, impact its efficacy, and jeopardize achieving VLS. Several participants shared their experiences, illustrating how the inadequate availability of food affects their ability to adhere to their treatment regimen.

*“I often take my medication without food, which weakens my body. I was once hospitalized because of this.”* (Adolescent 04, male, 17 years)

*“Sometimes, I have to take my medication when there’s no food available, so I have to take it on an empty stomach.”* (Adolescent 02, female, 16 years)

##### 3.4.1.4. Busy schedules

During the interviews, some ALHIV mentioned that their busy schedules, combining studies and income-generating activities, created constraints that made it difficult for them to take their medication regularly. These additional responsibilities can sometimes interfere with ART adherence to treatment schedules, as described by one ALHIV:

*“I often have to decorate for parties, and these tasks sometimes take longer than expected. This prevents me from getting home in time to take my medication.”* (Adolescent 23, boy, 16 years)

##### 3.4.1.5. Feeling healthy and not perceiving the necessity of ART

Like many chronic diseases, HIV can remain asymptomatic for a long period. Consequently, some PLHIV may feel well for months or even years without experiencing any immediate symptoms of their HIV status. This false sense of good health can cause them to underestimate the significance of ART. A member of the nursing staff exemplified this occurrence:

*“Some ALHIV, feeling healthy after taking medication for a while stop taking their treatment. This may be due to several factors: some do not understand that HIV infection is a chronic disease, whereas others, despite knowing their status, do not see the need to continue ART. However, this decision can have serious and unbearable long-term consequences for many patients.”* (FGD 04, HCW 02, female, doctor)

##### 3.4.1.6. Distance from home

Adolescents’ daily journeys, often far from home, complicate their medication routine, potentially compromising the effectiveness of ART and the achievement of VLS. Being away from home without their medications at hand can lead them to overlook the significance of consistent treatment.

*“On Sundays, I’m often very busy with lots of things to do. Sometimes I don’t get home until 8:00’ p.m., and as I don’t take my medication with me when I go to Mass, I end up forgetting to take it.”* (Adolescent 39, female, 17 years).

*“... when I went on holiday to stay with my uncle in Mbuji-Mayi, I stopped taking my medication…”* (Adolescent 25, male, 16 years)

#### 3.4.2. Interpersonal barriers: undisclosed HIV status.

Many ALHIV choose to keep their HIV status hidden from peers, sharing it only with their guardians and HCWs. This lack of disclosure creates challenges in adhering to ART, as it often leads to secrecy and stigma.

“I hide my medication from everyone except my aunt, as I don’t want others to know I’m sick.” (Adolescent 22, female, 18 years).

Caregivers also emphasized this challenge as a significant barrier to adherence to ART, underscoring the importance of ALHIV disclosure of their status for effective management.

*“Many caregivers are reluctant to openly address an adolescent’s HIV-positive status, often disguising the medication as treatment for a cough, a nutritional supplement like vitamins, or for another unrelated condition. However, upon discovering the truth, adolescents may react by resisting medication intake, thereby heightening their susceptibility to opportunistic infections from not achieving VLS.”* (FGD 02, HCW 07, female, nurse)

#### 3.4.3. Barriers at the family level.

##### 3.4.3.1. Broken families and parental loss

The family is central to an individual’s development; however, many ALHIV in this study reported challenges arising from parental loss, living with guardians, or broken families. These circumstances impede their ability to adhere to ART and achieve VLS.

*“Becoming an orphan at the age of 12, I moved in with my grandmother, who, due to her age, finds it challenging to take care of us. The loss of my parents, coupled with the enduring emotional distress, impacts my ability to adhere to my medication regimen consistently.”* (Adolescent 04, male, 17 years)

*“Since my parents passed away, my family stopped caring for me, except for my grandmother, which sometimes leads me to skip my medication.”* (Adolescent 26, male, 17 years)

##### 3.4.3.2. Lack of assistance in taking ART

HCWs emphasized that the lack of assistance in ensuring ALHIV take their medication is a significant barrier to ART adherence and achieving VLS. Some caregivers administer medications without verifying whether the adolescent actually consumes them, resulting in missed doses.

*“I’ve always advised ALHIV’s caregivers to be cautious, as some adolescents may deceive them by pretending to take their medication, holding it in their mouths, and then discarding it. I even witnessed the tragic outcome of a girl who deceived her grandmother by pretending to adhere to her medication, only to dispose of it when unsupervised. Owing to her irregular medication intake, the girl failed to achieve VLS despite multiple medical assessments, ultimately resulting in her untimely death.”* (FGD 02, HCW 03, male, pharmacist).

##### 3.4.3.3. Stigma and discrimination within the family

Family stigma and discrimination significantly impact ART adherence. ALHIV frequently face isolation and marginalization within their own homes, potentially resulting in treatment abandonment.

*“After my mother’s death, I faced discrimination at my aunt’s house. I was given separate dishes and cups, and no one would share with me. This mistreatment led me to stop taking my medication for two years.”* (Adolescent 04, male, 17 years)

#### 3.4.4. Barriers at the community level: stigma and discrimination.

Participants did not identify any community-level facilitators for achieving VLS but emphasized stigma and discrimination as major barriers to ART adherence and to achieve VLS. Within communities, social discrimination is widespread, especially towards young individuals with chronic illnesses such as HIV, often coming from peers or classmates. All ALHIV in this study mentioned hiding their HIV status to prevent stigmatization.

*“When my son’s father left me because of my HIV status, it discouraged me from continuing my medication. He claimed that if he hadn’t been infected yet, it was by chance, and he preferred to separate to avoid the HIV acquisition risk.”* (Adolescent 34, female, 19 years)

*“At home, I take my medication secretly. No one, including my friends at school, knows my status. This has caused major difficulties, like when I confided in a boy about my status and he left me.”* (Adolescent 37, female, 18 years)

## 4. Discussion

Achieving VLS among ALHIV is a significant public health challenge worldwide. While the context in Lubumbashi, DRC, reflects many of the issues faced in other settings, both in resource-limited and high-income countries, it highlights the universal factors impacting ART success among ALHIV. These challenges—such as poverty, stigma, lack of support, and issues related to medication adherence—are experienced by adolescents across the globe. Using the ecological model, this study identified specific barriers and facilitators to achieving VLS in Lubumbashi, revealing that both success and failure are influenced by multiple factors operating at various levels, including individual, family, interpersonal, health service, and community. These factors have been shown to either hinder or facilitate ART adherence and the achievement of VLS among ALHIV.

The higher proportion of adolescents aged 16-19 years (89.7%) compared to those aged 13-15 years (10.3%) in our study can be attributed to differences in availability during recruitment. Older adolescents are generally more independent and able to attend appointments on their own, whereas younger adolescents often rely heavily on their parents or caregivers to accompany them. This dependency may have limited the participation of younger adolescents, as their attendance was contingent on their caregivers’ availability and willingness to bring them to the study. This recruitment dynamic likely contributed to the age distribution observed in the sample. Participants in our study identified ART side effects, stigma, forgetting medication, lack of food, busy schedules, feeling healthy, travel, non-disclosure of HIV status, broken families, and lack of ART support as barriers to achieving VLS. In contrast, they identified confidence in ART efficacy, understanding ART’s importance, establishing a routine, using reminders, managing side effects, engaging in peer support, maintaining open relationships with healthcare staff, and being motivated for someone else’s sake as strategies for overcoming these barriers. These findings align with those from other qualitative studies identifying barriers and facilitators to achieve VLS. For instance, in Ghana [24] and Sierra Leone [25], healthcare staff support, parental involvement, disease knowledge, and the perception of positive outcomes were emphasized as motivators for PLHIV to achieve VLS. Izudi et al. [15] in Uganda noted the importance of understanding ART adherence, mental health support, peer support, personal reminders, and effective management of ART side effects. Similarly, Curioso et al. [26] in Peru highlighted the role of living for his/her loved ones, reminders from family, routine establishment, positive outcomes, and confidence in drug efficacy as essential facilitators of achieving VLS.

Forgetting medication, stigma, and financial barriers were key obstacles to ART adherence among PLHIV in Ghana and Ethiopia [24,27]. A Botswana study also highlighted ART side effects, lack of food, and financial constraints as barriers for ALHIV [28]. These findings align with ours and other studies [15,23]. In Peru, stigma, dietary challenges, busy schedules, and a sense of good health were identified as barriers to ART adherence [26]. This present study found that some ALHIV faced transportation difficulties, while others forgot medication due to religious activities, reflecting similar findings in Ethiopia [27]. This Ethiopian study showed that forgetting medication leads to unsuppressed VL [27], a phenomenon also observed in our study, where ALHIV experienced interruptions in ART due to busy social lives and school commitments. These findings underscore the necessity for interventions that target both practical and psychological barriers to enhance ART adherence and to achieve VLS.

Stigma and discrimination emerged as significant barriers to achieving VLS among ALHIV in our study, aligning with other studies identifying stigma as a major deterrent to ART adherence and VLS achievement. For instance, perceived stigma related to disclosure was highlighted as a key barrier among ALHIV in Ghana [24]. Interventions to reduce stigma and promote social inclusion are crucial for improving health outcomes in this population. Although community facilitators were not included in our study, prior research underscores their vital role in enhancing ART adherence. Community outreach programs have been shown to reduce stigma, provide support networks, and improve both adherence and the overall quality of life for ALHIV [29,30]. Most ALHIV in our study achieved VLS after receiving counseling and psychological support, particularly when encountering stigma. They also expressed gratitude for the positive relationships with healthcare staff. This underscores the significance of trust and open communication with HCWs, fostering a supportive environment for addressing HIV-related stigma and depression. These results are consistent with other studies that underscore the importance of strong relationships with HCWs and support systems, such as family reminders for medication, in achieving VLS [14,24].

In Lubumbashi, some ALHIV encountered difficulties in maintaining consistent ART intake due to medication side effects, such as gastrointestinal problems, headaches, and fatigue, which hindered ART adherence and VLS achievement. This aligns with prior studies linking concerns about side effects to suboptimal VLS outcomes, despite adherence counseling [31]. Particularly, gastrointestinal issues like nausea, loss of appetite, and diarrhea serve as major barriers to sustained treatment, affecting VLS [15,31].

The finding that a lack of food is a significant barrier to achieving VLS, especially for ALHIV who are orphans or from financially constrained households, highlights the importance of addressing basic nutritional needs as part of ART support. Consistent with previous research [1,15,32], this underscores the necessity of ensuring adequate food intake to improve ART adherence and, by extension, support VLS. Interventions should consider providing nutritional support or financial assistance to enhance medication adherence and prevent complications due to skipped doses. Addressing food insecurity is thus a critical component of comprehensive HIV care for ALHIV, as it directly impacts treatment outcomes and overall health.

This study identified a significant barrier to achieving VLS: the lack of parental supervision during ALHIV medication intake. Despite caregivers administering medication, many became preoccupied with other tasks and did not verify whether the medication was taken. Some ALHIV, facing challenges with their treatment, may conceal non-adherence by pretending to take the medication, which compromises achieving VLS. Similar to findings from a Peruvian study [26], our research also demonstrates that orphaned ALHIV or those from disrupted families encounter challenges that detrimentally impact ART adherence, thereby impeding VLS. The absence of parental support, coupled with emotional and financial instability, plays a role in non-adherence and suboptimal outcomes. Previous studies underscore the crucial importance of family in aiding long-term treatment adherence and offering essential psychosocial support to achieve VLS [23,24,33,34]. These findings collectively emphasize the vital role of family involvement and consistent supervision in ensuring ART adherence and achieving optimal outcomes for ALHIV.

The present study also revealed that many ALHIV were not adhering to ART correctly due to not being informed of their HIV-positive status. This lack of disclosure led to confusion, as some ALHIV thought they were managing a chronic cough rather than HIV. Disclosing HIV status, however, can improve the quality of life, social support, immune recovery, and ART adherence, while non-disclosure increases the risk of loss to follow-up and is a significant barrier to achieving VLS [15,35–37].

In addition, being away from home and having busy schedules were identified as significant barriers to achieve VLS, aligning with findings from other studies [1,26,27] showing that long travel distances and associated costs often result in missed appointments and ART non-adherence. Social support and participation in peer groups were identified as crucial facilitators of VLS, improving HIV knowledge and ART adherence through interpersonal interactions and peer support [1,15,26,29].

This study has several strengths, including a comprehensive exploration of barriers and facilitators to achieving VLS from the perspectives of ALHIV, their caregivers, and HCWs involved in ART adherence. Our sample size was substantial, and interviews continued until thematic saturation was achieved, providing a nuanced understanding of the factors affecting VLS. The application of the socioecological model to guide interview development, data analysis, and result presentation adds to the robustness and potential replicability of the study.

However, there are notable limitations. Firstly, the study’s urban focus may limit the generalizability of the findings to rural settings where socioeconomic conditions and HIV care quality differ. Additionally, the reliance on self-reported data introduces the possibility of social desirability bias, which could affect the accuracy of the responses. Furthermore, the study findings may not fully represent the experiences of ALHIV on second- or third-line ART regimens, as their experiences with ART and HIV control programs may vary.

## 5. Conclusion

This study, rooted in the socioecological model, highlights that barriers and facilitators to achieving VLS among ALHIV in Lubumbashi occur at multiple levels: personal, interpersonal, family, and community. Key barriers identified include drug side effects, stigma, forgetting to take medication, lack of food, busy schedules, feelings of being healthy, travel, nondisclosure of HIV status, broken families, and lack of assistance in taking ART. Conversely, confidence in drug efficacy, understanding the importance of ART, establishing a routine, using reminder tools, managing drug side effects, engaging in peer support clubs, maintaining open relationships with healthcare staff, and being motivated to stay well for loved ones were found to be critical facilitators.

To enhance VLS rates, HIV control programs ought to prioritize overcoming these barriers by optimizing drug management, reinforcing family and community support systems, and improving health services. Subsequent investigations should delve into inventive interventions aimed at ALHIV and their caregivers, along with tactics to diminish stigma and enhance ART adherence. Implementing practical measures, like broadening peer support initiatives, furnishing useful tools for medication adherence, and customizing counseling methods for ALHIV requirements, is crucial for sustained advancement. By capitalizing on the facilitators and addressing the obstacles uncovered in this study, programs can contribute to actualizing the global objective of achieving 95% VLS among ALHIV.

## Supporting information

S1 File The Supporting file contains three key documents: the Questionnaire Study, which details the questions used to collect data on barriers and facilitators to medication adherence, and viral load suppression; the Informed Consent Form, which provides parents or guardians with study details and seeks their consent; and the Child Assent Form, a simplified version for young participants (<18 years) to understand the study and give their agreement. These documents ensure ethical standards and participant protection.
(DOCX)

## Abbreviations

AIDS: Acquired Immunodeficiency Syndrome
ALHIV: adolescents living with HIV
ART: Antiretroviral Therapy
DRC: Democratic Republic of the Congo
FGD: focus group discussion
HCW: healthcare worker
HIV: Human Immunodeficiency Virus
IDI: in-depth interview
PLHIV: people living with HIV
SSA: Sub-Saharan Africa
UNAIDS: Joint United Nations Programme on HIV/AIDS
VL: viral load
VLS: viral load suppression
WHO: World Health Organization
