# Supplementary material for: Barriers and facilitators to HIV viral load suppression among adolescents living with HIV in Lubumbashi, Democratic Republic of the Congo: A qualitative study
Source: PLoS One. 2025 Mar 25;20(3):e0320417. doi: 10.1371/journal.pone.0320417 (PMC11936171; doi:10.1371/journal.pone.0320417)
Supplement: S1 File — These documents ensure ethical standards and participant protection. (DOCX) [file pone.0320417.s001.docx]

**QUESTIONNAIRE STUDY**

**Title of study: Barriers and facilitators to HIV viral load suppression among adolescents living with HIV in Lubumbashi, Democratic Republic of the Congo: A qualitative study.**

1. **Section A: Healthcare workers’ focus group discussion**
   1. **Healthcare workers’ characteristics**

- Age
- Sex
- Function in the HIV clinic care
  1. **Healthcare workers’ questions**
- How would you describe your experience working with adolescents with HIV?
- What are some of the health problems or opportunistic infections with which adolescents most often present?
- How often do adverse reactions to ARV occur?
- Do some of the adolescents with HIV you know have problems with adherence? What types of problems and why?
- When are the adolescents hospitalized?
- Have you experienced a time when two or more adolescents with HIV help each other?

1. **Section B: Parents/Caregivers (guardians)’ in-depth interview guide**
   1. **Parents/Caregivers (guardians)’ characteristics**

- Relationship to adolescents
- Age
- Sex
- Status HIV
- Occupation
- Marital status
- Educational level
  1. **Parents/caregivers questions**
- Has your adolescent learned to care for him/herself by, for example, taking his/her pills on time and eating well?
- What difficulties have you perceived that your son/daughter/charge has in managing his/her illness?
- Do you think that your son/daughter/charge knows to ask for help when s/he needs it?
- Would s/he be able to manage his/her own treatment?
- How do you help him/her take his/her ARV?

1. **Section C: Adolescents’ in-depth interview guide**
   1. **Characteristics of adolescents that participated in the psychosocial support groups**

- Sex
- Age
- HIV infection route : perinatal, transfusion, sexual, unknown
- Currently living with/in: At least one biological parent, A non-parent family member, Adoptive parents, Group housing, A non-family member.
- Death of at least one parent from HIV/AIDS
- Lifetime history of residence in group home for children with HIV/AIDS
- Main occupation
- Orphan status: non-orphan, single orphan, double orphan
- WHO HIV clinical stage
- Adherence status: adherent, non-adherent
  1. **Adolescent questions**

### Knowledge of HIV

Q1. Can you explain what you know about HIV/AIDS?
Q2. Could you share how you contracted the infection?

### General medication use

Q3. How many antiretroviral treatments (ART) are you currently on?
Q4. What are the names of the medications you're taking?
Q5. Can you tell me about when you first began taking these medications?
Q6. Are you taking any additional medications? How do you feel about the total number of medications you're on?
Q7. How did you feel during the first two weeks after starting your treatment?
Q8. Have you ever had your medication changed? If so, what was the reason for the change?
Q9. What do you think about your medication? Do you believe it's necessary?

### Viral load suppression and Adherence

Q10. Are you familiar with what viral load is and how it affects your health?
Q11. In your opinion, what factors influence the suppression of your viral load?
Q12. What challenges do you face in taking your medication regularly and keeping your viral load suppressed?
Q13. On which day(s) of the week do you most often miss taking your medication?
Q14. Why do you think that happens?
Q15. Can you describe your experience at home when it comes to taking your medication?
Q16. How do you manage taking your medication while at school?
Q17. What has been the most difficult experience you've had with your medication?

### Influence of others

Q18. How do you get along with the health workers at the clinic?
Q19. How is your relationship with the people who provide your medication?
Q20. How has this affected your attitude toward your medication?
Q21. How do you think living with or without your parents has influenced your ART management?
Q22. Do you talk about your HIV status with your friends or peers?
Q23. If not, what are the reasons behind that?
Q24. If yes, how has sharing your status impacted your commitment to taking your ARTs?

### Alcohol/drug use

Q25. Do you drink alcohol, smoke, or use drugs like crack or cocaine?
Q26. If so, have you experienced hangovers or other effects? How does this impact your regular ART intake?

### Conclusion

Q27. Is there anything else you’d like to share about your medications that we haven’t covered today?

*I have been asking you so many questions, is there anything you would like to ask me?*

*Thank you very much for your time.*

***Dr Olivier Mukuku***

***Telephone: +243997925649***

***Email: oliviermukuku@yahoo.fr***

***Institut Supérieur des Techniques Médicales de Lubumbashi, Democratic Republic of the Congo***

**INFORMED CONSENT FORM**

# **Title of study: Barriers and facilitators to HIV viral load suppression among adolescents living with HIV in Lubumbashi, Democratic Republic of the Congo: A qualitative study.**

***Principal investigator: Olivier K. Mukuku***

***Supervisors:***

1. ***Professor Kaymarlin Govender***
2. ***Professor Stanis O. Wembonyama***

**Ethical Clearance Protocol Number:** [N° HSSREC/00006817/2024]

**Medical Ethics Committee of the University of Lubumbashi**: [N° UNILU/CEM/036/2023]

Dear parent/guardian,

Good morning/afternoon. My name is Olivier Mukuku, and I am a student at the University of KwaZulu-Natal. As part of my studies, I am conducting research on HIV Viral Load Suppression. I would be grateful if you would consider participating in this study. Before you decide, you will be given detailed information about the study, have the opportunity to ask any questions, and receive a copy of this information sheet for your records.

We are inviting you to take part in a qualitative study into the experiences of adolescents living with HIV. The aim of this study is to gather valuable information about the challenges, needs and resources of adolescents living with HIV, in order to improve the support services available to them.

Before you decide to take part in this study, we ask you to read the information below carefully. Do not hesitate to ask questions if you need further clarification. Your participation is voluntary and you have the right to refuse or withdraw your consent at any time, without any negative consequences for you or your child.

The main aim of this study is to understand the experience of HIV-positive adolescents of adherence to antiretroviral treatment and viral load suppression. The information gathered will be used to improve support services and better meet the needs of adolescents living with HIV.

If you agree to take part, you will be invited to take part in a focus group with other parents/guardians of HIV-positive adolescents. Discussions will take place in a confidential and respectful environment. The focus group sessions will be recorded for later analysis, but the information gathered will be treated anonymously and confidentially. The interview will take approximately 45 to 60 minutes. We will make every effort to ensure that the interview is conducted at a time and place that is convenient and comfortable for you.

Your anonymity and the confidentiality of your answers will be strictly respected. The data collected will be used solely for research purposes and will not be disclosed to any unauthorized third party. Audio recordings will be stored securely and only members of the research team will have access to them.

Your participation in this study will contribute to a better understanding of the needs of HIV-positive adolescents and to improving the services available to them. There are no major risks associated with your participation, but you may feel emotionally affected by sharing your experience.

Your participation in this study is voluntary. You have the right to refuse to participate or to withdraw your consent at any time, without any negative consequences for you or your child. Your decision to participate or not will have no impact on the healthcare services you or your child receive.

By signing this form, you confirm that you have read and understood the information provided above. You consent to your participation in the study and to the use of the information collected for research purposes.

Name of parent/guardian: _______________________

Signature of parent/guardian: _______________________

Date : _______________________

Signature of researcher: _______________________

Date : _______________________

Please keep a copy of this form for your personal records.

Thank you very much for your participation and your valuable contribution to this study.

If you have any further questions or would like more information, please contact:

***Dr Olivier Mukuku Telephone: +243997925649***

***Email: oliviermukuku@yahoo.fr***

***Institut Supérieur des Techniques Médicales de Lubumbashi, Democratic Republic of the Congo***

**CHILD ASSENT FORM**

**Title of study: Barriers and facilitators to HIV viral load suppression among adolescents living with HIV in Lubumbashi, Democratic Republic of the Congo: A qualitative study.**

***Principal investigator: Olivier K. Mukuku***

***Supervisors:***

1. ***Professor Kaymarlin Govender***
2. ***Professor Stanis O. Wembonyama***

**Ethical Clearance Protocol Number:** [N° HSSREC/00006817/2024]

**Medical Ethics Committee of the University of Lubumbashi**: [N° UNILU/CEM/036/2023]

Dear [Child's Name],

Good morning/afternoon. My name is Olivier Mukuku, and I am a student at the University of KwaZulu-Natal. As part of my studies, I am conducting research on HIV Viral Load Suppression. I would be grateful if you would consider participating in this study. Before you decide, you will be given detailed information about the study, have the opportunity to ask any questions, and receive a copy of this information sheet for your records.

We would like to invite you to participate in our research study, which aims to understand the challenges and factors that affect HIV viral load suppression among adolescents in the Congo. We believe that your insights and experiences are crucial to helping us improve the well-being of HIV-positive adolescents.

**Purpose of the study:** The purpose of this study is to explore the barriers and facilitators that Congolese HIV-positive adolescents face in achieving viral load suppression. Your participation will help us gather important information to improve support and healthcare services for adolescents living with HIV.

**Your role:** If you agree to participate, you will be asked to share your thoughts and experiences in a confidential and supportive interview setting. We will respect your privacy and ensure that your identity remains confidential throughout the study.

**Voluntary participation:** Your participation in this study is entirely voluntary. You have the right to refuse to participate or withdraw from the study at any time without facing any negative consequences. Your decision will not affect the care or services you receive.

**Confidentiality:** Your personal information will be kept confidential. Any information shared during the study will be anonymized, and only the research team will have access to it. We will not use your name or any identifying information in our reports.

**Duration of the interview:** The interview will take approximately 30 to 45 minutes. We will make every effort to ensure that the interview is conducted at a time and place that is convenient and comfortable for you.

**Benefits:** While there may not be direct benefits to you, your participation will contribute valuable insights to the improvement of healthcare services for adolescents living with HIV. Your contribution may help enhance the support available to others facing similar challenges.

**Risks:** The study involves discussing personal experiences related to HIV. This may be emotionally sensitive. We will have a qualified and experienced counselor available for support during and after the interview.

### **Additional consent for future research**

In addition to your participation in this study, we would like to ask your permission to use the data collected from you for future research projects. These projects will be related to the current study but may explore different aspects of HIV care and support for adolescents. Your data will remain confidential, and your identity will not be disclosed in any future research.

**Please indicate if you agree to allow your data to be used for future research:**

- Yes, I agree to the use of my data for future research: _______ (Initials)
- No, I do not agree to the use of my data for future research: _______ (Initials)

**Contact Information:** If you have any questions or concerns about the study, please feel free to contact Dr Olivier Mukuku (Tel: +243997925649).

**Permission:** Before you decide whether or not to participate, we encourage you to discuss this study with your parent/guardian. We will also seek their permission for your involvement. Your parent/guardian may be present during the consent process.

I, the undersigned, have read and understood the information provided in this Child Assent Form. I agree to participate voluntarily in the study.

**Child's Name (Print):______________________**

**Child's Signature:_________________________**

**Date:______________________**

Parent/Guardian Consent: I, the undersigned, am the parent/guardian of the child named above. I have read and understood the information provided in this Child Assent Form. I give permission for my child to participate in the study.

**Parent/Guardian Name (Print):______________________**

**Parent/Guardian Signature:_________________________**

**Date:______________________**

***Dr Olivier Mukuku***

***Telephone: +243997925649***

***Email: oliviermukuku@yahoo.fr***

***Institut Supérieur des Techniques Médicales de Lubumbashi, Democratic Republic of the Congo***
